# Dendritic spine head diameter predicts episodic memory performance in older adults

**DOI:** 10.1126/sciadv.adn5181

**Published:** 2024-08-07

**Authors:** Courtney K. Walker, Evan Liu, Kelsey M. Greathouse, Ashley B. Adamson, Julia P. Wilson, Emily H. Poovey, Kendall A. Curtis, Hamad M. Muhammad, Audrey J. Weber, David A. Bennett, Nicholas T. Seyfried, Christopher Gaiteri, Jeremy H. Herskowitz

**Affiliations:** ^1^ Department of Neurology, Center for Neurodegeneration and Experimental Therapeutics, University of Alabama at Birmingham, Birmingham, AL 35294, USA.; ^2^ Rush Alzheimer’s Disease Center, Rush University Medical Center, Chicago, IL 60612, USA.; ^3^ Department of Biochemistry, Emory University School of Medicine, Atlanta, GA 30322, USA.; ^4^ Department of Psychiatry, SUNY Upstate Medical University, Syracuse, NY 13210, USA.

## Abstract

Episodic memory in older adults is varied and perceived to rely on numbers of synapses or dendritic spines. We analyzed 2157 neurons among 128 older individuals from the Religious Orders Study and Rush Memory and Aging Project. Analysis of 55,521 individual dendritic spines by least absolute shrinkage and selection operator regression and nested model cross-validation revealed that the dendritic spine head diameter in the temporal cortex, but not the premotor cortex, improved the prediction of episodic memory performance in models containing β amyloid plaque scores, neurofibrillary tangle pathology, and sex. These findings support the emerging hypothesis that, in the temporal cortex, synapse strength is more critical than quantity for memory in old age.

## INTRODUCTION

Episodic memories define the past from the present and provide humans with a recollection of personal experiences. Deficits in episodic memory are observed following injury to the temporal cortex, and these cognitive functions decline at varying degrees as humans age or experience neurodegenerative disease (*1*–*3*). Dendritic spines serve as the postsynaptic compartment for most excitatory synapses in the brain, and synapse strength is inseparably linked to spine morphology. Spine loss occurs naturally throughout mammalian aging in brain regions that are critical for memory processes (*4*, *5*). In Alzheimer’s disease (AD), loss of spines or synaptic markers correlates more strongly with memory impairment than β amyloid (Aβ) plaques or neurofibrillary tangles (NFTs) (*6*–*9*). These findings have collectively fueled the long-standing hypothesis that decreased density of spines or synapses accounts for the decline in memory abilities among older adults. However, spines are lost through the natural course of aging (*4*, *5*, *10*). Therefore, we hypothesized that there must be features of dendritic spines or synapses that contribute to memory ability in old age that are distinct from the natural progression of spine loss or the accumulation of common age-related neurodegenerative pathologies, like Aβ plaques. Thus, we sought to identify features that predict episodic memory function in old age.

## RESULTS

### Dendritic spines were sampled and analyzed from the frontal and temporal cortex

**Fig. 1.**
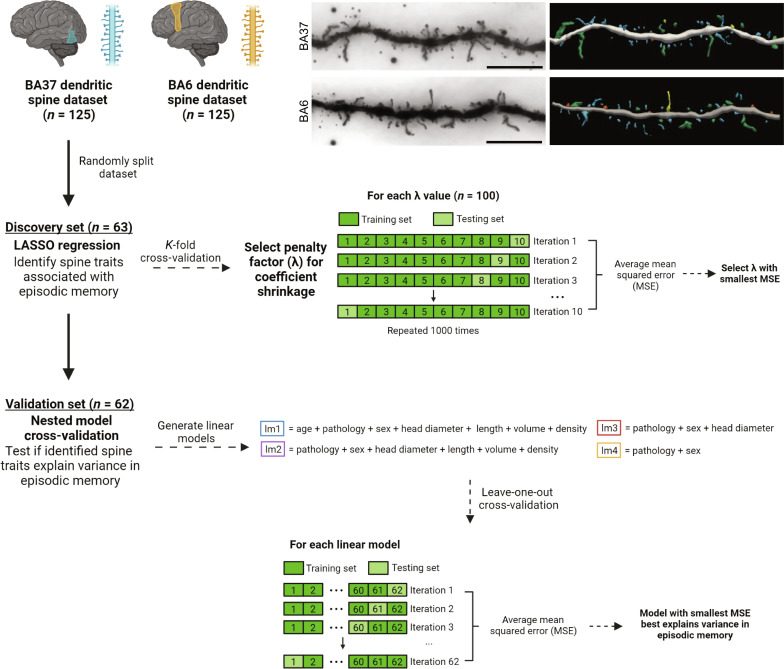
Overview of workflow. Sections (250 μm) of the BA37 temporal cortex and BA6 premotor cortex from 128 older adults were Golgi stained and imaged with high-resolution bright-field microscopy. Z-stacks were imported to Neurolucida 360 for 3D digital reconstruction. Representative 60X bright-field images of Golgi-stained dendrites in BA37 and BA6 of cognitively normal older adults are shown on the right with the digital 3D reconstructions of those segments. Scale bars, 10 μm. Blue, thin spines; orange, stubby spines; green, mushroom spines; yellow, filopodia. Three cases were removed from downstream analyses due to missing data, resulting in a final sample size of 125. BA37 and BA6 datasets were used separately. Each dataset was randomly split into a discovery set containing 63 participants and a validation set containing 62 participants. LASSO regression was performed on the discovery set to identify spine traits associated with episodic memory. *K*-fold cross validation (*K* = 10) was used to select the penalty factor used for coefficient shrinkage during LASSO regression. Nested model leave-one-out cross-validation was then performed using the validation set. Multiple linear models containing different combinations of variables (age, pathology scores, sex, and spine density, head diameter, length, and volume) were generated. Pathology scores included were NP score and NFT score. The model with the smallest MSE best predicted episodic memory.

Postmortem samples of Brodmann area (BA) 6 superior frontal gyrus within the premotor cortex and BA37 inferior temporal gyrus (ITG) within the temporal cortex were obtained from 128 individuals from the Religious Orders Study and Rush Memory and Aging Project (ROSMAP). Cases were 90.53 ± 6.06 (mean ± SD) years of age, with variable cognitive performance scores and AD-related neuropathology. To measure dendritic spine density and morphology, BA37 and BA6 tissue slices were imaged at 60X using bright-field microscopy with a high–numerical aperture (NA) condenser. Z-stacks were reconstructed in three dimensions using Neurolucida 360 (Fig. 1 and fig. S1). All dendritic spine data are provided in tables S1 and S2.

### Dendritic spine head diameter in the temporal cortex improves model prediction of episodic memory scores

BA37 and BA6 dendritic spine datasets were individually run through a supervised learning algorithm (*11*) to determine if specific dendritic spine features dictate episodic memory performance beyond the effect of other traits, including AD-related neuropathology (Fig. 1). Both dendritic spine datasets were split into a discovery set (*n* = 63) and a validation set (*n* = 62); three cases were removed due to missing scores of AD pathology and/or memory performance. Least absolute shrinkage and selection operator (LASSO) regression was performed on the discovery set to determine which dendritic spine features were most strongly associated with episodic memory function. First, the penalty factor (λ) was selected by performing 1000 iterations of 10-fold cross-validation for 100 values of λ. The penalty factor was then applied to each variable during LASSO regression to calculate the coefficient for each variable, which indicates the relative contribution of each dendritic spine feature to episodic memory performance. The validation set was used to confirm the results of LASSO regression by performing nested model leave-one-out cross-validation. Multiple models for predicting episodic memory were tested to determine which combination of variables best predicts episodic memory function. Last, correlations between dendritic spine parameters and cognitive and pathology scores were calculated using the complete dataset for each brain region individually.

**Fig. 2.**
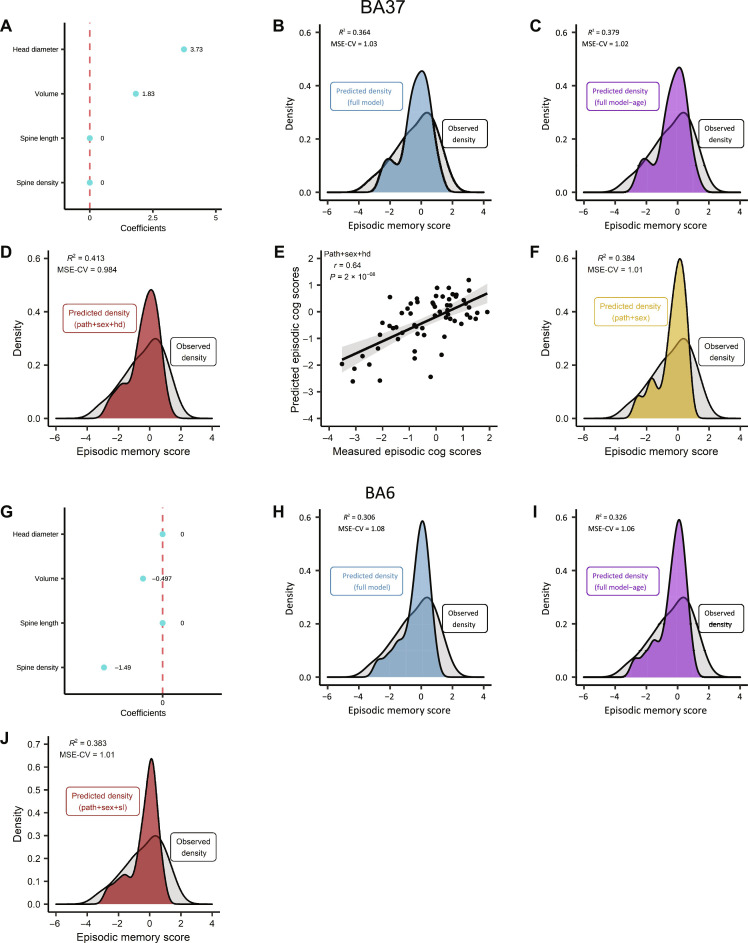
Dendritic spine head diameter improves the model prediction of episodic memory scores. (**A**) LASSO coefficients after shrinkage for each BA37 dendritic spine feature included in the regression. Of the four spine features, head diameter had the strongest association with episodic memory. (**B**) Density plot comparing the observed episodic memory scores with those predicted by the full model consisting of pathology scores, sex, age, and BA37 dendritic spine density, length, head diameter, and volume. (**C**) Density plot comparing the observed episodic memory scores with those predicted by a model containing all features except age. Removing age improves model prediction. (**D**) Density plot comparing the observed episodic memory scores with those predicted by a model containing pathology scores (path), sex, and BA37 dendritic spine head diameter (hd). This model provided the best prediction of episodic memory scores. (**E**) Pearson correlation between the observed episodic memory scores and those predicted by the best model (pathology, sex, and BA37 head diameter). Cog, cognitive. (**F**) Density plot comparing the observed episodic memory scores with those predicted by the model containing pathology scores and sex. (**G**) LASSO coefficients after shrinkage for each BA6 dendritic spine feature included in the regression. Of the four spine features, BA6 spine length had the strongest association with episodic memory. (**H**) Density plot comparing episodic memory scores with the full model, consisting of pathology scores, sex, age, and BA6 dendritic spine density, length, head diameter, and volume. (**I**) Density plot comparing episodic memory scores with a model containing all features except age. Removing age improves model prediction. (**J**) Density plot comparing episodic memory scores with a model containing pathology scores, sex, and BA6 dendritic spine length (sl). The model fit (*R*^2^) is slightly lower than the model in (F), which contains only pathology scores and sex as predictors.

In the discovery set, LASSO regression identified BA37 spine head diameter as having the strongest association with episodic memory score (Fig. 2A and fig. S2, A and B). Nested model cross-validation was then used to replicate the regression model in a separate validation set by comparing linear models. The full model contained age; measures of neuritic Aβ plaques and NFTs; sex; and spine density, length, head diameter, and volume (Fig. 2B). Age was included on the basis that cognitive decline occurs with age (*12*). Aβ plaques and NFTs accumulate with age and AD progression (*13*) and can influence memory function. Sex was included in the model because, in this sample, males were found to harbor significantly less neuritic Aβ plaques and NFTs than females (fig. S2, C and D).

Linear models containing different combinations of variables were generated. Removing age from the full model increased the model fit (Fig. 2C). The model fit was also improved by removing spine density, length, and volume (Fig. 2, D and E). The model containing neuritic Aβ plaque and NFT pathology scores, sex, and dendritic spine head diameter best predicted episodic memory performance in comparison to all other models. Without head diameter, the model (pathology + sex) provided a less accurate prediction of episodic memory scores (Fig. 2F). Sex contributes to the prediction of episodic memory scores, given the reduction in model fit for the model containing only pathology scores and spine head diameter (figs. S2, E and F, and S7).

Next, BA6 dendritic spine density, length, head diameter, and volume were included in a LASSO regression model to determine which BA6 spine features associated with episodic memory performance. In the discovery set, LASSO regression identified BA6 spine length as having the strongest association with episodic memory score (Fig. 2G and fig. S3, A and B). However, the association was smaller than what was observed for BA37 spine head diameter. Nested model cross-validation was then used to replicate the BA6 LASSO regression model in a separate validation set by comparing linear models. Removing age from the full model improved the prediction of episodic memory (Fig. 2, H and I). However, in contrast to the models containing BA37 spine traits, the addition of BA6 spine parameters reduced model performance (Fig. 2J). These models were also compared by estimating empirical mean squared error (MSE) confidence intervals using a bootstrapping procedure (fig. S4). Thus, the contribution of dendritic spine head diameter to episodic memory performance is specific to the BA37 temporal cortex in these datasets.

### Dendritic spine head diameter in the temporal cortex positively correlates with episodic memory

**Fig. 3.**
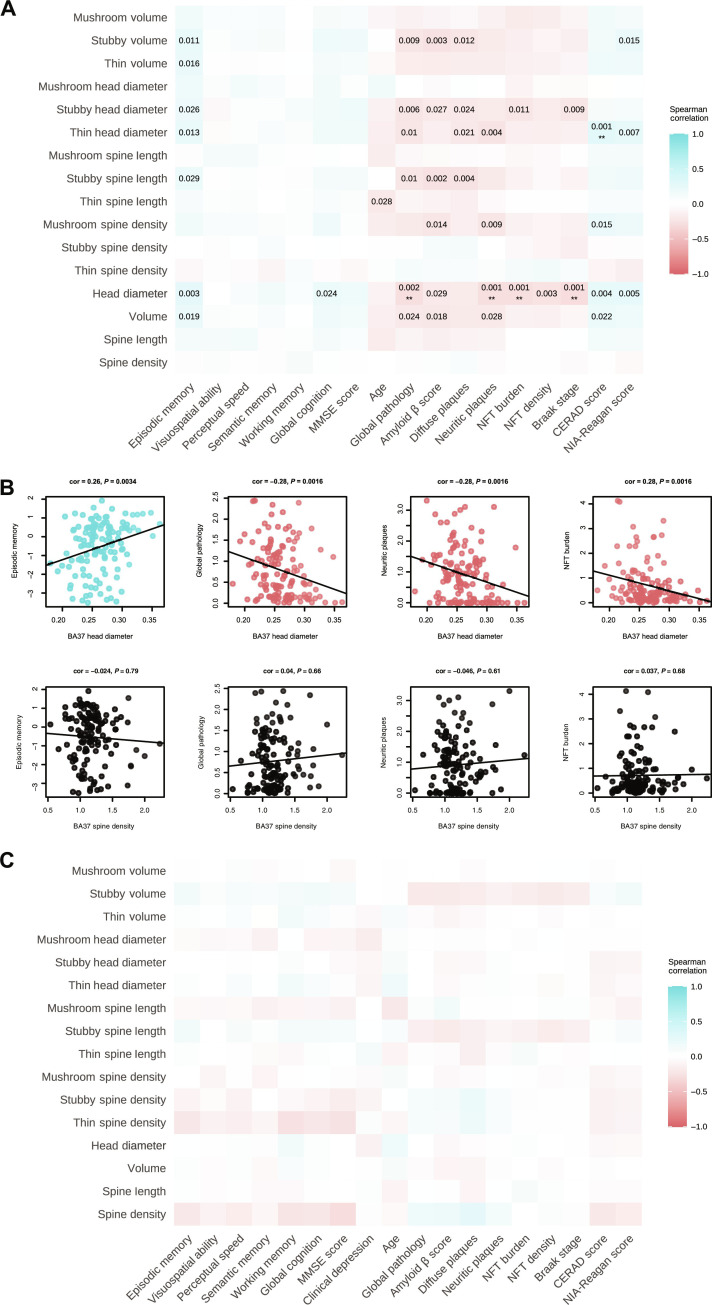
Dendritic spine morphology correlates with cognition and pathology in BA37 but not BA6. (**A**) The heatmap depicts Spearman correlations between BA37 dendritic spine measurements and both cognitive test scores and pathology scores. An FDR of 10% (*q* < 0.1) was applied, and the unadjusted *P* value is shown for those correlations with *P* < 0.05 and *q* < 0.1. Correlations with *P* < 0.05 and *q* < 0.05 following FDR adjustment are indicated with double asterisks (**). (**B**) Representative Spearman correlation (corr) scatter plots for BA37. (**C**) Spearman correlations between BA6 dendritic spine measurements and both cognitive test scores and pathology scores are shown in the heatmap. No correlations were statistically significant (*P* < 0.05) following FDR adjustment (*q* < 0.1).

Last, we assessed the relationship between BA37 dendritic spine features and cognitive scores and AD-related pathology measures by performing Spearman correlations using the entire BA37 dataset (*n* = 125). Episodic memory score was correlated significantly with many BA37 dendritic spine features. Mean spine volume and mean head diameter measurements, as well as volume and head diameter for individual spine subclasses, exhibited significant correlations with episodic memory score (Fig. 3, A and B and fig. S5). The positive correlation between spine head diameter and episodic memory score remained significant at a false discovery rate (FDR) of 5%. Notably, BA37 spine density was not significantly correlated with cognitive scores or AD-related pathology traits. No significant correlations were observed between BA6 spine features and cognition scores or AD-related pathology measures (Fig. 3C).

## DISCUSSION

Our goal was to evaluate spine density and morphology with respect to episodic memory from at least two different brain regions, one in the frontal cortex and one in the temporal cortex. From a practical standpoint, appropriately sized frozen tissue samples from both BA6 and BA37 were available for all 128 ROSMAP participants that were included in this study, making it possible to conduct the experiments. Functional magnetic resonance imaging (fMRI) is commonly used to measure brain activity while individuals reason the relation between items based on episodic memory abilities. Several fMRI studies conducted in this manner indicate activity in both BA37 and BA6 (*14*, *15*), including older individuals with mild cognitive impairment (*16*). BA37 contains portions of the fusiform face area, which is critical for facial recognition in humans and retrieval of episodic memory (*17*–*19*). In addition, activity in BA6 was measured by fMRI during episodic memory encoding and recognition in amnestic mild cognitive impairment, cognitively normal older adults, and patients with AD (*20*, *21*). While activity in both BA37 and BA6 are implicated in fMRI studies that evaluate episodic memory, future studies examining the spine morphology in other brain regions, including the hippocampus and prefrontal cortex, could yield informative data.

A previous study demonstrated that acquisition learning in aged nonhuman primates was inversely correlated with thin spine volume in the dorsolateral prefrontal cortex (*22*), which is somewhat contrasting to the findings herein. Therefore, the positive correlation between spine head diameter and episodic memory in BA37 may not be generalizable to other brain regions or other forms of memory. Notably, our previous work in the BA46 dorsolateral prefrontal cortex revealed a reduction in thin spine head diameter among cognitively normal older apolipoprotein E (APOE) ε4 allele carriers (*4*); however, including the APOE genotype within the models herein did not improve predictability (fig. S6). When integrated with the findings from Dumitriu *et al.* (*22*), we posit that structural remodeling of thin spines in the prefrontal cortex is critical for maintenance of information processing and memory circuits in aging (*6*, *23*). Collectively, we hypothesize that different mechanisms of dendritic spine remodeling would be expected to preserve specific types of memory, based on the brain region, in aging or under the threat of AD or other neurodegenerative pathologies.

Dendritic spine head diameter scales with the size of the postsynaptic density and number of α-amino-3-hydroxy-5-methyl-4-isoxazolepropionic acid receptors (AMPARs), reflecting functional synaptic strength (*24*). Gamma oscillatory activity (30 to 80 Hz) and long-term potentiation (LTP) are associated with formation and retrieval of episodic memories (*25*). During LTP, gamma oscillations play an important role in spike timing–dependent plasticity, whereby spines enlarge, forming the structural basis of LTP (*26*). Through these mechanisms, it is possible that older adults with larger spine head diameters in the temporal cortex exhibit better episodic memory performance due to the strength of the remaining synapses rather than the number of synapses.

Notably, several BA37 spine morphologic features were significantly correlated with measures of Aβ pathology, more so than NFTs. Experimental model systems indicate that Aβ induces dendritic degeneration of neurons, including spine loss, which causes detrimental hyperexcitability by rendering neurons more electrically compact (*27*). This phenotype leads to aberrant circuit synchronization and subsequent cognitive impairment in human amyloid precursor protein (hAPP) mice and likely in patients with AD (*28*–*33*). Regarding spine morphology, elegant studies that used iontophoretic microinjection of Lucifer yellow (LY) followed by high-resolution confocal laser scanning microscopy and dendritic three-dimensional (3D) reconstructions for morphometry analysis revealed that Aβ drives reductions in both hippocampal spine density and spine length, predominantly affecting thin spines in 6-month-old hAPP mice (*34*) and mushroom spines in 12-month-old APP Dutch mice (*35*). Comparatively in humans, we found inverse correlations between BA37 mean spine head diameter and Aβ scores as well as mushroom spine density and Aβ scores (Fig. 3, A and B, and fig. S5). The consistency in effects on mushroom spine density among humans and mice may be due to the older age of the APP Dutch mice that were evaluated by Price *et al.* (*35*). The apparent lack of effects on spine head diameter in APP mice could be due to brain region–specific or species-specific neurobiology. However, an obvious confound is that Aβ-induced effects on spines in APP mice are studied in isolation, whereas a majority of older humans harbor both Aβ and tau pathology, especially in the temporal lobe. Yet, it is curious that, in the BA6 superior frontal gyrus, where Aβ is the predominant pathology (*36*), we did not observe correlations between Aβ measures and spines. From a mechanistic standpoint, one hypothesis is that Aβ oligomers interact with the cellular prion protein (PrP^C^) (*37*), inducing PrP^C^-mediated signaling of RhoA guanosine triphosphatase (*38*). Activation of RhoA and one of its primary downstream effectors, Rho-associated protein kinase (ROCK) isoform 2, can cause collapse of dendritic architecture, including spine loss (*34*, *39*–*41*). ROCKs regulate actin cytoskeleton rearrangement (*42*–*46*), and increased activity of ROCK2 in AD likely causes detrimental consequences on dendritic spine morphology and structural plasticity (*34*, *47*).

Regarding tau, one general hypothesis is that pathologic tau induces synapse silencing without causing overt destruction of dendritic spines (*48*). Silent synapses lack functional AMPARs, rendering the synapse inactive (*49*, *50*). The vulnerability of AMPARs to tau pathology can drive synaptic dysfunction in age-related tauopathies (*51*–*53*), and while tau accumulation in dendritic spines can reduce neuronal activity and availability of surface AMPARs, tau does not alter synaptic density in in vitro models (*54*). Moreover, tau does not induce spine loss in primary hippocampal neuron cultures or organotypic slices (*54*–*56*). However, a confound to these studies is that a majority of these findings used expression of human tau with familial frontotemporal dementia mutations; therefore, comparisons with AD are tentative.

In a previous work, we compared dendritic spine density and morphology among pyramidal neurons in the mouse medial prefrontal cortex using 3D digital reconstructions of (i) bright-field microscopy z-stacks of Golgi-impregnated dendrites and (ii) confocal microscopy z-stacks of LY fluorescent dye–filled dendrites (*57*). Notably, LY microinjection enables measurement of spine density and morphology similar to that observed by electron microscopy (*58*); however, this method is nearly impossible to use with brain-banked postmortem human tissue samples (*10*). We found that spine volume measurements were larger using Golgi staining compared to LY microinjection, whereas head diameter was comparable for Golgi staining and LY microinjection (*57*). The difference in volume may be due to differences in axial resolution of bright-field and confocal microscopy (*59*). Therefore, it is possible that spine volume measurements using Golgi staining in human tissue samples may be less accurate than head diameter, and spine volume should not be ruled out as a contributor to episodic memory.

Past studies by Scheff *et al.* in 2011 demonstrated that estimates of the total number of synapses in ITG are reduced in amnestic mild cognitive impairment and AD compared to no cognitive impairment (*60*). These findings are highly consistent with our previous work measuring the dorsolateral prefrontal cortex and entorhinal cortex spine density in controls and AD cases (*6*, *61*). Scheff *et al.* also showed there were positive correlations between the number of synapses and Mini-Mental Status Examination (MMSE) and verbal fluency, seemingly across all participants. Last, Scheff *et al.* (*60*) demonstrated that the total synapse estimates in ITG were inversely correlated to age. In the study herein, we found no correlations between spine density and cognitive traits. The mean age of participants in (*60*) was 86.4 ± 6.8 years, whereas the mean age of participants in this study was 90.53 ± 6.06. One possibility is that, due to the older age of individuals in our study, the natural loss of spines in aging had mostly taken effect in ITG BA37, leaving the remaining spines to cope with memory tasks (*4*, *5*, *10*). In this scenario, increases in spine head diameter could be a critical compensatory mechanism to maintain synaptic connections and fuel cognitive processes, which has been previously posited (*62*, *63*). Another possibility is that spine density may not be an accurate measure of total synapses. For instance, an unknown number of spines measured by Golgi staining in our study could be silent and lack integration with functional synapses, therefore not contributing to memory processes (*64*).

Our findings herein suggest a potential utility for positron emission tomography (PET) ligands that indicate spine head diameter to predict impending memory impairment or serve as a biomarker for future therapeutics. As an example, changes at the receptor level can be detected by PET imaging, as evidenced by the observation of reduced metabotropic glutamate subtype 5 receptors availability in the hippocampus of patients with AD (*65*). On the basis of this, a PET ligand for AMPARs could potentially serve as a marker of spine head diameter or synaptic strength; however, the presence of extra-synaptic AMPARs could complicate this approach. Moreover, this idea would only be feasible once PET reaches higher resolutions in the distant future. To explore this idea, we conducted Pearson correlations on multiplex tandem mass tag mass spectrometry proteomic data (*n* = 7787 proteins) that were previously generated on BA37 postmortem brain tissue samples from 115 of the same cases used in this study (*36*). We tested for associations between individual protein abundance levels and spine head diameter (table S3). Among the 30 most correlated proteins to spine head diameter, we did not observe AMPARs or AMPAR-related proteins. However, the top protein, ranked by the highest *r* correlation magnitude, was muskelin, which binds directly to γ-aminobutyric acid A receptor α1 (GABA_A_R α1) and undergoes cotraffic with GABA_A_R α1 (*66*). In this way, muskelin facilitates endocytosis of GABA_A_R through associations with myosin and dynein motor complexes (*66*). However, whether the muskelin protein level or function is altered in aging or AD remains to be determined.

We observed that older adults with larger dendritic spine head diameters in the temporal cortex exhibited better episodic memory performance, with no effect on dendritic spine density. Targeting pathways that maintain spine head diameter or synaptic strength, rather than pathways that maintain or generate new spines or synapses, could potentially yield greater therapeutic benefits for older adults in preclinical phases of AD.

## MATERIALS AND METHODS

### Human participants

Postmortem samples of BA6 and BA37 were obtained from the ROSMAP (*67*). Participants enroll without known dementia. All agree to annual clinical evaluation and brain donation at death. They were approved by an institutional review board of Rush University Medical Center. Participants signed informed consent, an Anatomical Gift Act, and a repository consent to allow their resources to be shared. Cases exhibited an array of brain pathologies and cognitive scores, as detailed in table S1. Appropriately sized frozen tissue samples from both BA6 and BA37 were available for all participants in this study, making it possible to conduct the experiments.

ROSMAP participants underwent cognitive testing, including tests of episodic memory, visuospatial ability/perceptual orientation, perceptual speed, semantic memory, and working memory. All scores are composite scores or averages of the *Z* scores calculated for each cognitive test used to assess each realm of cognitive function. The episodic memory score was assessed by seven cognitive tests; visuospatial ability/perceptual orientation by two tests; perceptual speed by four tests; semantic memory by three tests; and working memory by three tests. A global cognition score was generated by averaging *Z* scores for 19 cognitive tests, encompassing those used to assess each realm of cognitive function. Further details on cognitive testing were described by Folstein *et al.* (*68*). In addition to these cognitive tests, the MMSE was also administered to assess cognitive function in ROSMAP participants. A clinical diagnosis of major depressive disorder was rendered by an examining physician at last visit prior to death (*69*). The clinical depression diagnosis was based on criteria of the Diagnostic and Statistical Manual of Mental Disorders, 3rd edition, revised (DSM-III-R), clinical interview with the participant, and review of responses to a series of questions adapted from the Diagnostic Interview Schedule. Annual evaluations are performed for cognitive assessments, and participants are contacted quarterly to determine vital status and changes in health. Hence, the interval of most recent cognitive assessment and day of death, when the autopsy was performed, is consistent among all participants within a 12-month time frame (*70*).

The diffuse plaque score, neuritic plaque (NP) score, and NFT burden were generated by examining silver-stained diffuse and NPs and NFTs across the entorhinal cortex, hippocampus, midtemporal cortex, inferior parietal cortex, and midfrontal cortex. Global pathology burden is derived from these diffuse plaque, NP, and NFT counts. Aβ load, percent area occupied by image analysis, was determined by immunohistochemistry for Aβ across the hippocampus, entorhinal cortex, midfrontal cortex, inferior temporal cortex, angular gyrus, calcarine cortex, anterior cingulate cortex, and superior frontal cortex. NFT density by stereology was based on AT8 tau staining across the same eight regions. The semiquantitative Consortium to Establish a Registry for Alzheimer’s Disease (CERAD) score ranges from 1 to 4: 1 = definite AD, 2 = probable AD, 3 = possible AD, and 4 = no AD, based on NP load. Braak staging was categorized by the distribution and severity of Bielschowsky silver-stained NFTs. The National Institute on Aging (NIA)–Reagan score uses both Braak staging and CERAD score to generate a postmortem diagnosis of AD, ranging from 1 to 4: 1 = high likelihood of AD, 2 = intermediate likelihood of AD, 3 = low likelihood of AD, and 4 = no AD. More information on the pathology scores can be found in (*67*).

### Golgi-Cox staining

Golgi-Cox staining was used to visualize dendrites and dendritic spines in human brain samples from BA6 and BA37 using the FD Rapid Golgi Stain Kit (FD Neurotechnologies, catalog no. PK401), with adjustments. All of the following steps were performed at room temperature. Solutions A (potassium dichromate and mercuric chloride) and B (potassium chromate) were combined 48 hours prior to tissue submersion. A 2-ml solution A+B was placed in each well of a 12-well plate (Fisher Scientific, catalog no. 08-772-29). Frozen tissue blocks, approximately 10 mm x 10 mm x 10 mm, were immediately dropped into the A+B solution. The chromate solution was replaced after 24 hours, and the tissue blocks were incubated in the solution for exactly 21 days. On day 21, the tissue blocks were moved to a new 12-well dish containing solution C, which was replaced after 24 hours. After 72 total hours in solution C, each tissue sample was sliced in 125-μm sections in solution C using a Leica Vibratome (VT1000 S). Free-floating sections were placed in a six-well dish (Fisher Scientific, catalog no. 353046) containing solution C. A free-floating method was used for staining; sections were sequentially moved from solution D, solution E, and distilled water according to the manufacturer’s instructions. Slices were dehydrated with alcohols (70, 90, and 100% ethanol) and cleared with xylenes (Fisher Scientific, catalog no. X3P). Slices were then placed on glass slides (Fisher Scientific, catalog no. 12-550-15) with one slide spacer (Electron Microscopy Sciences, catalog no. 70327-20S), sealed with Permount (Fisher Scientific, catalog no. SP15-100), and coverslipped with 0.13- to 0.17-mm-thick glass (24 x 50 mm; Carolina Biological, catalog no. 633153). Slides were dried in the dark for at least 1 week prior to imaging.

### Bright-field microscopy

Dendrites in BA6 and BA37 were imaged by a blinded experimenter. Each case had multiple slides available for imaging. From each tissue section, one to two pyramidal neurons from layer 2 or 3 were imaged for analysis. Between 8 and 12 neurons were sampled per individual. A single dendritic segment was imaged per neuron. Dendrite segments were imaged if the following criteria were met: (i) located centrally within the total tissue depth, (ii) not obscured by staining debris and/or intersecting neighboring neurons, and (iii) fully impregnated. In addition, only secondary dendrites that were over 30 μm in length, not a distal tip, and had an approximate diameter of 1 μm were imaged. Each tissue slice was visualized under a 10X magnification to determine tissue quality and region(s) of interest. Once a quality region was established, the tissue was viewed at a 60X magnification using Type F immersion oil (Nikon, catalog no. MXA22168). Z-stacks were captured at a step size of 0.1 μm at 60X using a Nikon Plan Apo 60X/1.40 NA oil-immersion objective in combination with a high NA (NA = 1.4) oil condenser (Nikon, catalog no. MEL51420, Ti2-C-LHO HNA Condenser Lens) on a Nikon Eclipse Ti2 inverted microscope with a Lumencor SOLA light engine and Hamamatsu ORCA-flash 4.0 digital camera. Each image was 1024 x 1024 pixels.

### 3D digital image reconstruction

Reconstructions of dendrites and dendritic spines were conducted by a blinded experimenter. ND2 files were converted to 16-bit TIFF files in ImageJ and then imported to Neurolucida 360 (MBF Biosciences, version 2.70.1). Dendritic segments were traced using a user-guided semiautomated directional kernel algorithm. Initiation and termination points for dendrite reconstruction were established using the following criteria: (i) ≥5 μm away from the distal tip of the dendrite, (ii) consistent diameter, (iii) level axis with limited smear in the *z* plane, and (iv) ≥20 μm in length. Once the dendritic branch was traced, the experimenter verified that the points located on the dendrite were accurate in *X*, *Y*, and *Z* planes and made manual adjustments if necessary. The diameter of the dendrite at each point was also verified to ensure accuracy. Dendritic spines were traced using voxel clustering. We used the following parameters for spine identification: outer range, 7 μm; minimum height, 0.3 μm; detector sensitivity, 90 to 125%; and minimum count, 8 voxels. The morphology of each reconstructed spine was examined to verify that axial smear did not cause misrepresentation, and the merge and slice tools were used to correct inaccuracies. The position of each spine backbone point was confirmed by the experimenter. To correct a misrepresentative backbone, the spine was viewed from the *Z* plane, and experimenters moved backbone points in the *X*-*Y* plane. Any repositioning in the *X*-*Z* or *Y*-*Z* plane was performed while the spine was being viewed from the lateral angle. Morphometric analysis was conducted for each spine, and measurements categorized spines into thin, stubby, mushroom, and filopodia classes. For spine classification, the following established parameters were used: head-to-neck ratio, 1.1; length-to-head ratio, 2.5; mushroom head size, 0.35 μm; and filopodium length, 3.0 μm. Spines with a head-to-neck ratio of >1.1 and head diameter of >0.35 μm were classified as mushroom. Spines were classified as filopodia or thin if the head-to-neck ratio was <1.1 and either (i) the length-to-head ratio was >2.5 or (ii) the head size was <0.35 μm. Of these, if the total length was >3.0 μm, the spine was classified as filopodia, and if <3.0 μm, as thin. Spine volume was measured by the number of voxels that make up the spine object multiplied by the volume of a single voxel. The volume of a single voxel is *X* resolution x *Y* resolution x *Z* resolution.

Reconstructions were exported to Neurolucida Explorer (MBF Biosciences, version 2.70.1), where data were collected for quantitative analysis. The dendritic spine measurement parameters included spine length and spine head diameter, among others. These parameters were exported and collected in Microsoft Excel. Derived measurements, such as spine density, were calculated from raw measurement data. Spine density was calculated by determining the number of spines per 10 μm of dendrite length. Spine length was defined as the curvilinear backbone length from the insertion point to the most distal point of the spine head. Head diameter was defined as the breadth of the spine head at its widest cross-sectional point. Both morphological measurements and corresponding backbone reconstructions were verified.

In total, we analyzed 45,763 μm of dendrite length (BA6: 22,660 μm; BA37: 23,103 μm) from 2157 neurons (BA6: 1072; BA37: 1085). Spine morphometry data were collected from 55,521 individual spines (BA6: 28,341; BA37: 23,103).

### Statistical analysis

#### Overview

We used a multistage approach to validate the generalizability and replicability of our results (*71*). A similar approach was used in a recent study that compared brain morphological features and reasoning performance (*11*). BA37 and BA6 dendritic spine data were considered as two separate datasets. For both datasets, three samples were removed due to missing data, for a final sample size of *n* = 125. For each dataset, samples were split via random sampling (seed 111) into a discovery sample (*n* = 63) and a replication sample (*n* = 62). Using the discovery sample, we implemented LASSO regression on overall BA37 or BA6 dendritic spine traits to determine which morphological features contributed most significantly to episodic memory performance in older adults. LASSO regression allows for a data-driven discovery procedure by inducing a penalty factor (λ) that shrinks unimportant coefficients with regard to the response variable (episodic memory score) (*72*). This shrinkage of coefficients via regularization enforces sparsity, improves the generalizability of results, accounts for multicollinearity, and decreases variance (*71*). All models were fit with cross-validation to optimize performance within the sample. Last, we replicated our own results in a separate replication sample (*n* = 62). To demonstrate that the spine traits selected by LASSO are generalizable, we compared nested linear models in the replication sample to show that the same dendritic spine traits selected by LASSO also improve the prediction of episodic memory score. In the replication sample, we include covariates for age, sex, and neuropathology in the nested linear models to demonstrate that dendritic spine traits have an effect beyond those explained by the covariates. Repeated 10-fold cross-validation and leave-one-out cross-validation were used for LASSO and nested linear model comparison, respectively. For all analyses, seed 111 was set in R (version 4.1.0) for replicability.

#### LASSO regression

LASSO regression can determine which spine traits are most strongly associated with episodic memory performance. Four dendritic spine traits were included as predictors in the model: density, length, head diameter, and volume. LASSO regression imposes an L1 norm, which enforces minimization of the product of a shrinking parameter (λ) and the absolute value of the coefficients (β). This results in small coefficients shrinking to zero and produces sparse models that are easier to interpret. LASSO regression selects important features through model simplification. For our application, the coefficients of the dendritic spine traits shrink until an optimal model with the strongest predictors remain. We used 10-fold cross-validation to select the parameter λ, per recommendations from its developers and by convention (*72*). R (version 4.1.0) and the R package glmnet were used to perform a grid search of 100 λ values to pinpoint the value that minimized cross-validated MSE (MSE-CV). To account for potential instability, this cross-validation procedure was repeated 1000 times and the median of the λ values was used to produce the final model.

#### Nested model cross-validation

To validate our results, we compared nested models to predict the episodic memory score using the replication sample. NP score and NFT burden were included in the model as measurements of pathology. Sex was included in the model because NP score and NFT burden differed by sex in our sample. In addition, age was included in the model. Because the models were nested, the *R*^2^ and MSE-CV of each model were compared to the others to assess fit and prediction error. Leave-one-out cross-validation was used to fit the nested models because this type of cross-validation has low bias and variance when used to compare nested models (*73*).

#### Spearman correlations

Correlations between dendritic spine features and pathology or memory scores were performed using Spearman correlations. Multiple comparisons were controlled with Storey’s *q* value at an FDR of 10% (*q* < 0.1). A more stringent FDR of 5% (*q* < 0.05) was applied, as indicated in the figures with asterisks. Heatmaps were generated using R (version 4.1.0) and the R packages ggplot2 and qvalue.
