# Supplementary material for: Dendritic spine head diameter predicts episodic memory performance in older adults
Source: Sci Adv. 2024 Aug 7;10(32):eadn5181. doi: 10.1126/sciadv.adn5181 (PMC11305389; doi:10.1126/sciadv.adn5181)
Supplement: Supplementary file 1 — Figs. S1 to S7 Legends for tables S1 to S3 [file sciadv.adn5181_sm.pdf]

Science Advances
AAAS

Supplementary Materials for

Dendritic spine head diameter predicts episodic memory performance in older adults

Courtney K. Walker *et al.*

Corresponding author: Jeremy H. Herskowitz, jhersko@uab.edu

Sci. Adv. **10**, eadn5181 (2024)
DOI: 10.1126/sciadv.adn5181

The PDF file includes:

Figs. S1 to S7
Legends for tables S1 to S3

Other Supplementary Material for this manuscript includes the following:

Tables S1 to S3

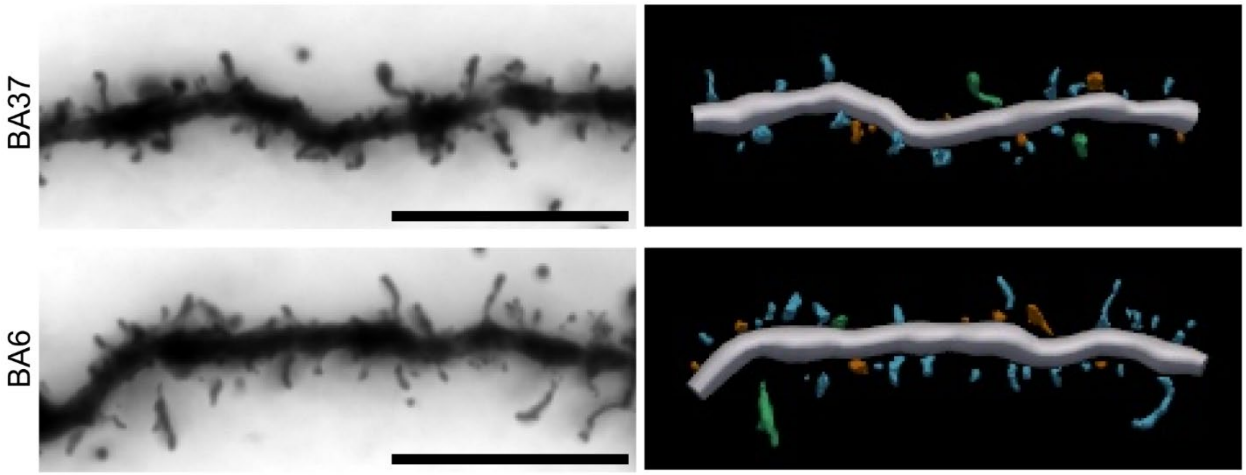

Supplementary Figure 1. Representative images. Representative 60X brightfield images of Golgi-stained dendrites in BA37 and BA6 of cognitively normal older adults are shown on the left. Scale bars = 10 µm. The digital three-dimensional reconstructions of those segments are shown on the right. Blue = thin spines, orange = stubby spines, green = mushroom spines.

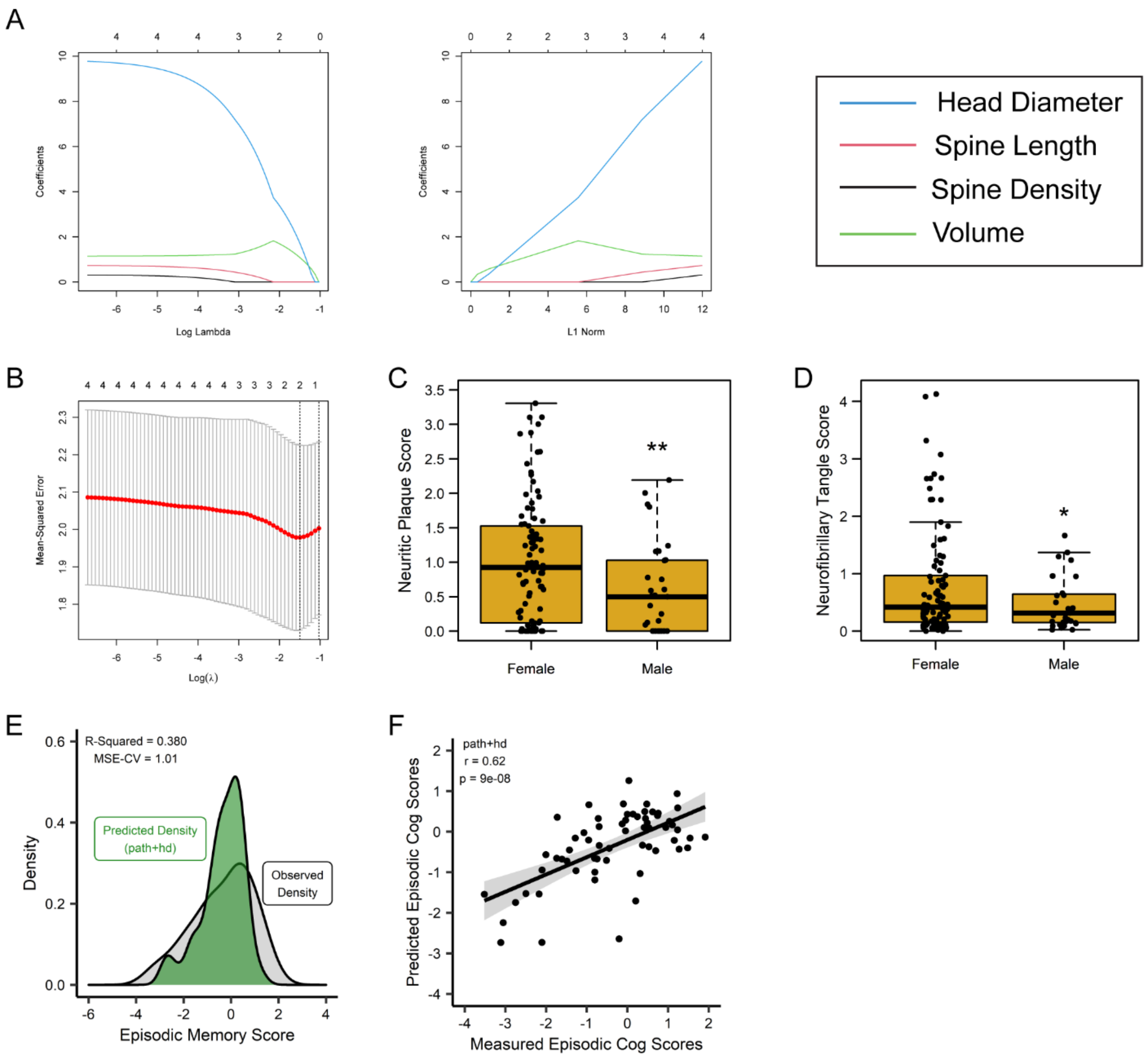

Supplementary Figure 2. LASSO regression and nested model cross-validation for BA37. (A-B) Example of one run of penalty factor (lambda) selection. Plot of coefficient for each log lambda value for each dendritic spine feature. Blue is head diameter, green is volume, red is length, and black is spine density. This procedure was repeated 1000 times, and we take the median lambda for 1000 lambdas that generated the respective model minimum mean square error (MSE) for the actual LASSO that is reported in the main text. The left plot shows shrinking of the coefficients at increasing values of log lambda where the numbers on top correspond to the number of non-zero coefficients remaining. The right plot shows increasing of the "maximal permissible value of the L1 norm" which is a part of the loss function that is used to mathematically fit LASSO regression. As the maximal permissible value gets larger, coefficients enter the model. **(B)** This plot shows the mean square error (MSE) of the model over the grid search of 100 lambda values for each 10-fold cross validation. The first dotted line represents the log lambda value for that run that corresponds to the lowest MSE model. **(C)** Neuritic plaque score is lower in males than females. Welch two sample t-test ($t(67.955) = 2.7114$, $p = 0.00848$). **(D)** NFT score is lower in males than females. Welch two sample t-test ($t(106.43) = 2.5479$, $p = 0.01226$). **(E)** Density plot

of episodic memory scores predicted by a model containing only pathology scores and head diameter overlaid on the plot of observed episodic memory scores. **(F)** Pearson correlation between the observed episodic memory scores and those predicted by a model containing pathology scores and head diameter.

A

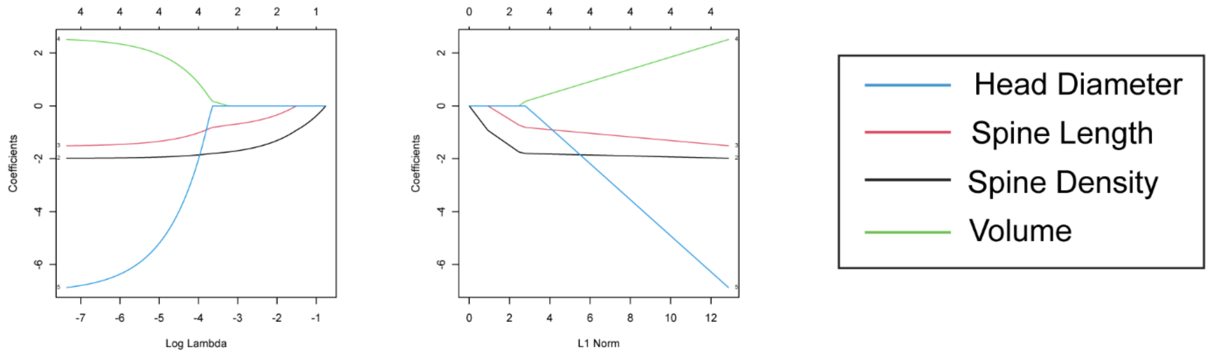

B

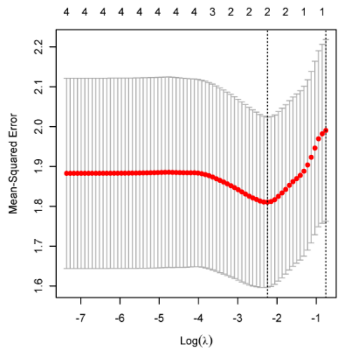

Supplementary Figure 3. LASSO regression and nested model cross-validation for BA6. (A-B) Example of one run of penalty factor (lambda) selection. Plot of coefficient for each log lambda value for each dendritic spine feature. Blue is head diameter, green is volume, red is length, and black is spine density. This procedure was repeated 1000 times, and we take the median lambda for 1000 lambdas that generated the respective model minimum mean square error (MSE) for the actual LASSO that is reported in the main text. The left plot shows shrinking of the coefficients at increasing values of log lambda where the numbers on top correspond to the number of non-zero coefficients remaining. The right plot shows increasing of the "maximal permissible value of the L1 norm" which is a part of the loss function that is used to mathematically fit LASSO regression. As the maximal permissible value gets larger, coefficients enter the model. **(B)** This plot shows the mean square error (MSE) of the model over the grid search of 100 lambda values for each 10-fold cross validation. The first dotted line represents the log lambda value for that run that corresponds to the lowest MSE model.

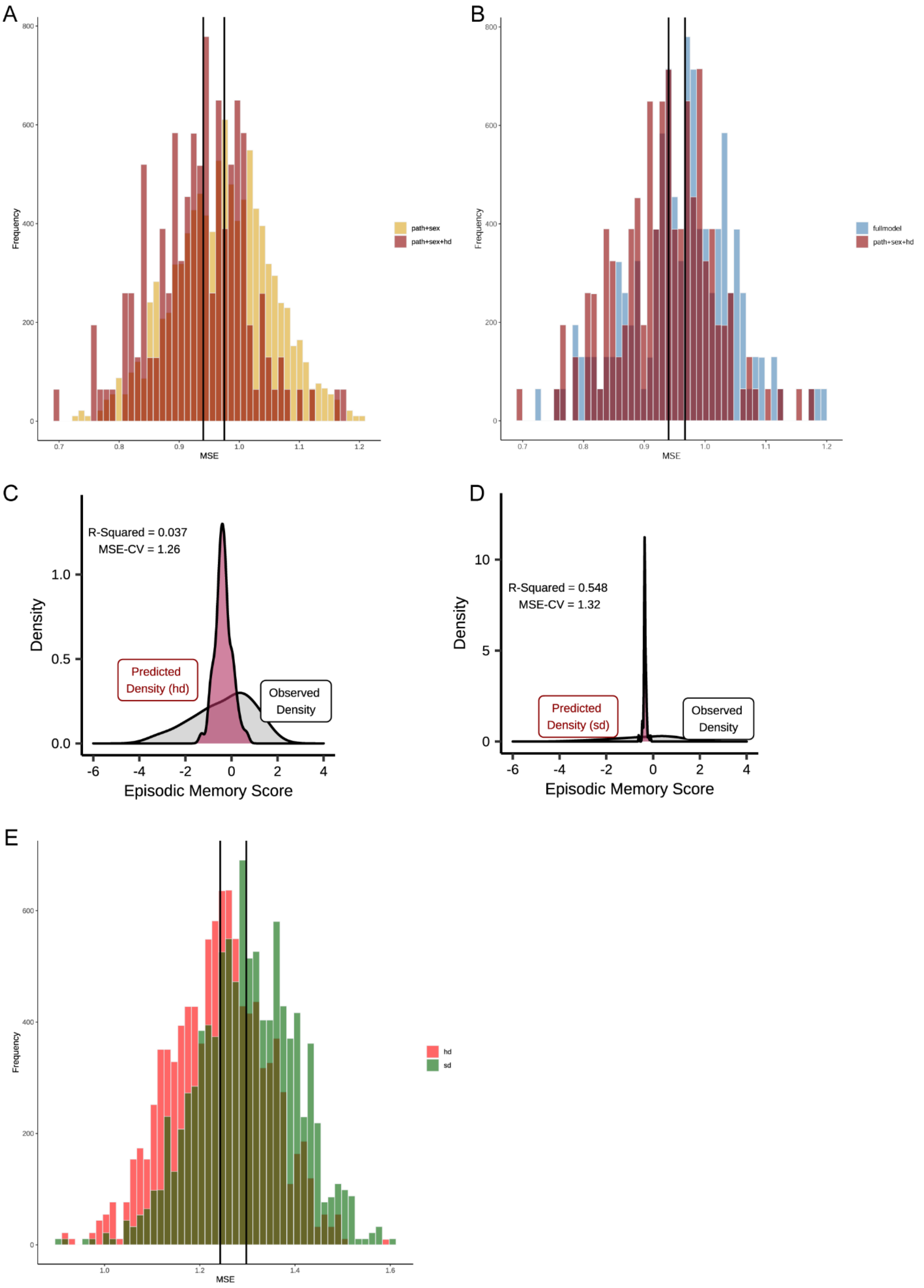
A
Frequency
MSE
path+sex
path+sex+hd
B
Frequency
MSE
fullmodel
path+sex+hd
C
Density
R-Squared = 0.037
MSE-CV = 1.26
Predicted Density (hd)
Observed Density
Episodic Memory Score
D
Density
R-Squared = 0.548
MSE-CV = 1.32
Predicted Density (sd)
Observed Density
Episodic Memory Score
E
Frequency
MSE
hd
sd

Supplementary Figure 4. Estimation of MSE confidence intervals using a bootstrapping procedure. We compared the full model, path+sex model, and path+sex+hd model by estimating empirical mean square error (MSE) confidence intervals using a bootstrapping procedure. Code was rewritten natively in R version 4.1.0. Leave-one-out cross-validation (looCV) was repeated over n=10000 iterations for comparing MSE-bootstrap of nested linear models, where the resample n is equivalent to the number of samples in the validation set (n=62). We report the 95% confidence intervals and medians of the MSE-CV-bootstrap of the full model, path+sex, and path+sex+hd. We also include the comparison of the full model versus pathology+sex+hd. Histograms are provided to compare the **(A)** path+sex and path+sex+hd models as well as to compare the **(B)** full model and path+sex+hd. Fullmodel: (Bootstrapped 95% $CI_{MSE} = 0.7831 - 1.1263$, $median_{MSE} = 0.9784334$). Path+sex model: (Bootstrapped 95% $CI_{MSE} = 0.8073 - 1.1119$, $median_{MSE} = 0.9646683$). Path+sex+hd model: (Bootstrapped 95% $CI_{MSE} = 0.7622 - 1.1001$, $median_{MSE} = 0.9399909$). Density plots for nested linear models, using spine head diameter or spine density alone to predict episodic memory were generated. The density plots are MSEcv-bootstrap histograms comparing the model for **(C)** head diameter (hd) and the model for **(D)** spine density (sd). Confidence intervals of MSEcv-bootstrap are reported at 95%. Spine head diameter (hd) model: (Bootstrapped 95% $CI_{MSE} = 1.0274 - 1.4414$, $median_{MSE} = 1.239907$). Spine density (sd) model: (Bootstrapped 95% $CI_{MSE} = 1.0584 - 1.5111$, $median_{MSE} = 1.292606$). While the R^2 for spine density alone is high, the MSEcv is greater than head diameter alone. We interpret this as spine density exhibiting lesser predictive value for episodic memory in comparison to head diameter, following bootstrap. These findings are supported by the correlation heatmap in Figure 3, demonstrating that spine head diameter associates significantly with several cognitive and pathology measures, while spine density does not. Comparing the R^2 and MSE of the head diameter only model to the best model, worse performance on prediction of episodic memory is observed. Hence, while the addition of head diameter measurements improves prediction, it does not perform better than pathological measures and sex on its own. **(E)** A histogram is provided to compare the model for hd and the model for sd.

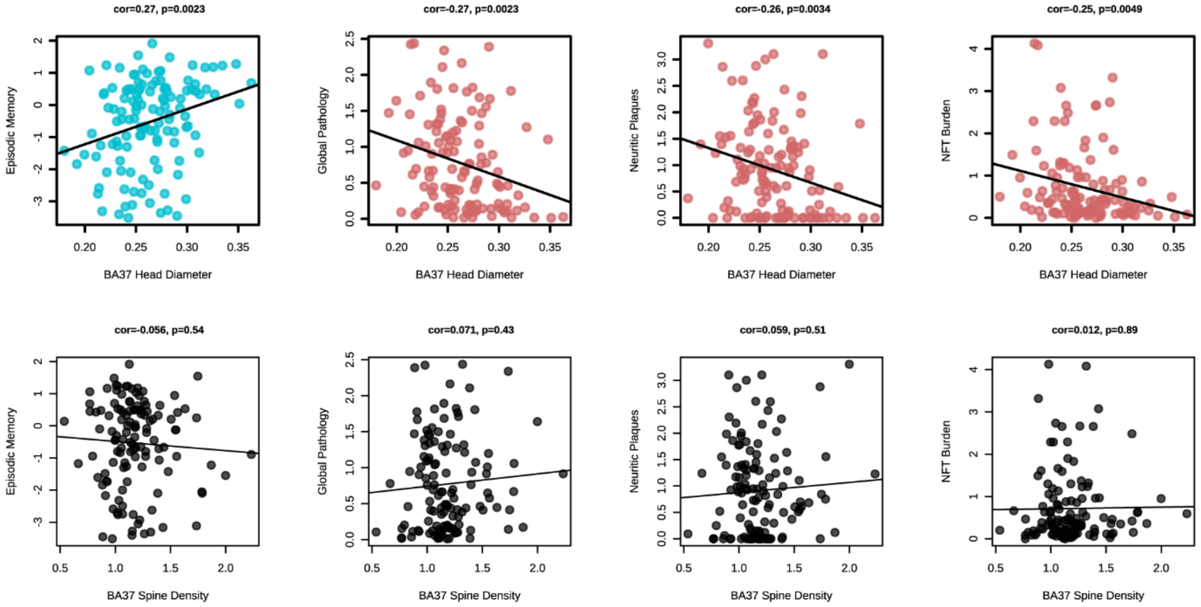

Supplementary Figure 5. Representative Pearson correlation plots for BA37. Pearson correlations were performed on BA37 spine head diameter or spine density and episodic memory, global pathology, neuritic plaque scores, and neurofibrillary tangle (NFT) burden. Results are highly similar to Spearman correlations displayed in Figure 3A-B.

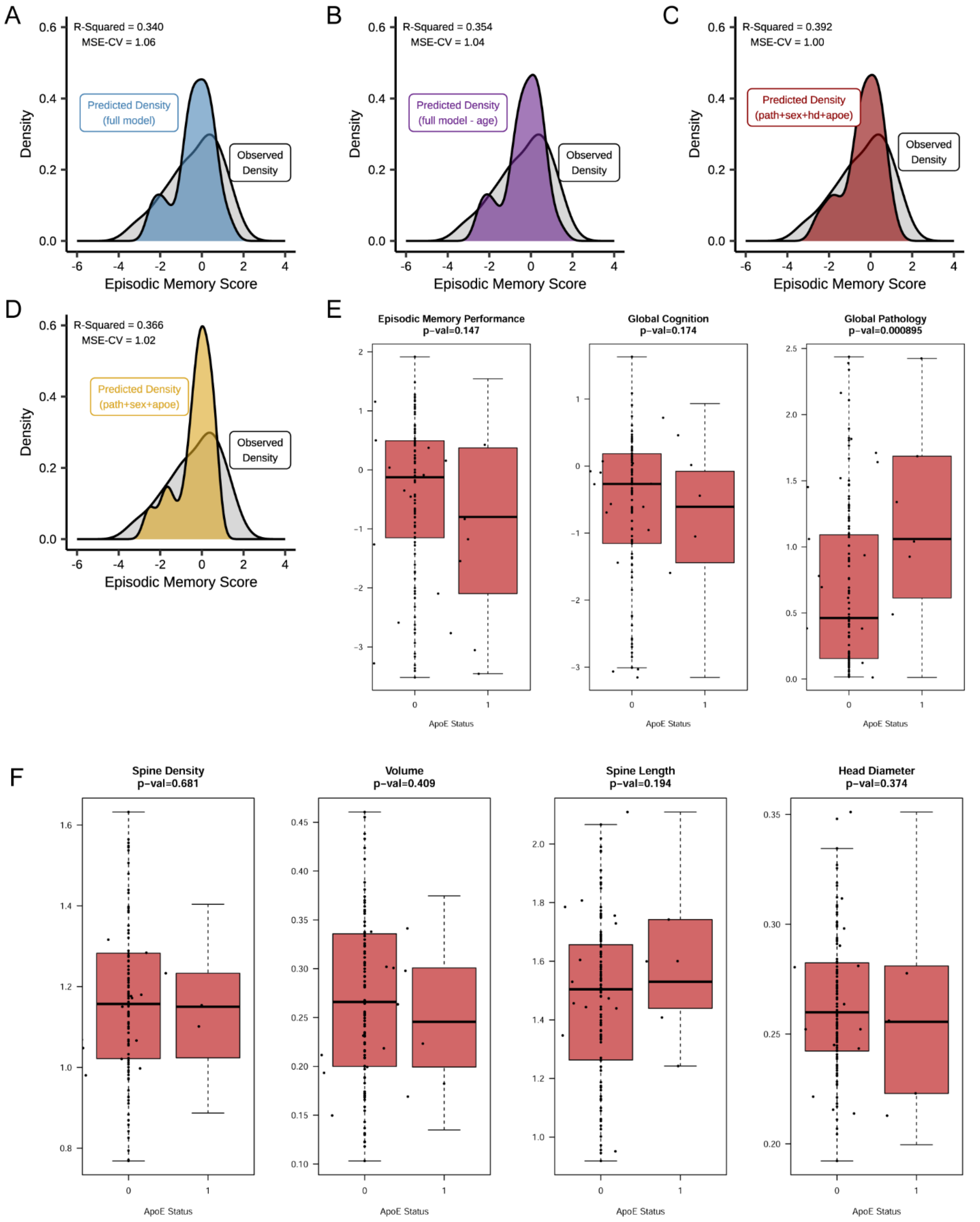

Supplementary Figure 6. APOE4 status does not improve model prediction of episodic memory scores. The array of APOE genotypes amongst the 128 individuals in the sample set were dichotomized to indicate the presence or absence of an APOE4 allele. This generated a group of

98 individuals without an APOE4 allele and 25 with an APOE4 allele. Five individuals did not have recorded APOE genotype status and were left out of the analysis. Maintaining the random splitting via index values of row in the manner of our other analyses revealed only 10 individuals within the validation set that harbor an APOE status of 1 (+4 allele). (A) Density plot comparing the observed episodic memory scores with those predicted by the full model consisting of pathology scores, sex, age, APOE4 status, and BA37 dendritic spine density, length, head diameter, and volume. (B) Density plot comparing the observed episodic memory scores with those predicted by a model containing all features except age. (C) Density plot comparing the observed episodic memory scores with those predicted by a model containing pathology scores, sex, APOE4 status, and BA37 dendritic spine head diameter. This model provided the best prediction of episodic memory scores. (D) Density plot comparing the observed episodic memory scores with those predicted by the model containing pathology scores, sex, and APOE4 status. (E) Non-parametric Wilcoxon rank sum test boxplots to assess univariate differences of +/- APOE4 status and episodic memory performance, global cognition, and global pathology. Box-and-whisker plots with overlaid jittered strip chart shows upper extreme, upper quartile, median, lower quartile, and lower extreme. The jittered points show distribution of the data points on top of the box-and-whisker and potential outlier. These plots are drawn for episodic memory performance, global cognition, and global pathology (Y-axis measures) by APOE status split by 0 and 1 (0 coding for no presence of the APOE4 allele and 1 coding for presence of the APOE4 allele) (X-axis). P-values reflect the statistical significance of Wilcoxon-rank-sum test. (F) Non-parametric Wilcoxon rank sum test boxplots to assess univariate differences of +/- APOE4 status and BA37 dendritic spine density, volume, length, and head diameter. Box-and-whisker plots with overlaid jittered strip chart shows upper extreme, upper quartile, median, lower quartile, and lower extreme. These plots are drawn for spine density, volume, spine length, and spine head diameter by APOE status split by 0 and 1 (0 coding for no presence of the APOE4 allele and 1 coding for presence of the APOE4 allele). P-values reflect the statistical significance of Wilcoxon-rank-sum test.

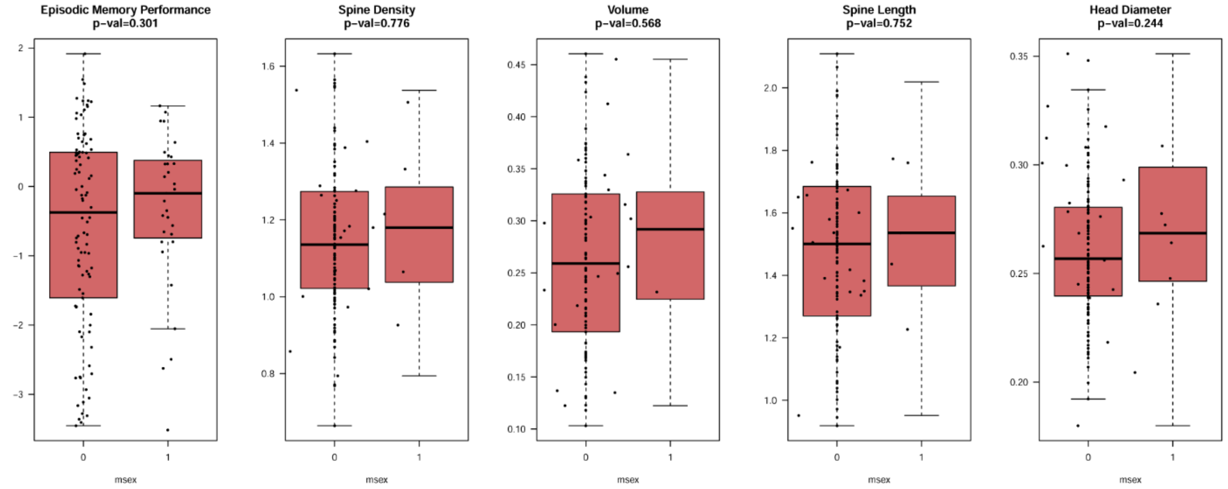

Supplementary Figure 7. Statistical analyses of sex and episodic memory, spine density, volume, length, and head diameter. Box-and-whisker plots with overlaid jittered strip chart shows upper extreme, upper quartile, median, lower quartile, and lower extreme. Plots are drawn for episodic memory performance, spine density, spine volume, spine length, or spine head diameter (Y-axis measures) by sex split by 0 and 1 (0 coding for female and 1 coding for male) (X-axis measures). P-values reflect the statistical significance of Welch two-sample t-test.

Tables S1 to S3

Table S1. BA37 spine data.

Table S2. BA6 spine data.

Table S3. BA37 proteins associated with spine head diameter.
